# Ethical, Legal and Social Issues of Digital Phenotyping as a Future Solution for Present-Day Challenges: A Scoping Review

**DOI:** 10.1007/s11948-021-00354-1

**Published:** 2021-12-20

**Authors:** Ana Tomičić, Anamaria Malešević, Anto Čartolovni

**Affiliations:** 1 Digital Healthcare Ethics Laboratory (Digit-HeaL), Catholic University of Croatia, Ilica 242, 10000 Zagreb, Croatia; 2 School of Medicine, Catholic University of Croatia, Ilica 242, 10000 Zagreb, Croatia

**Keywords:** Digital phenotyping, ELSI, Digital health, healthcare, health policy, Embedded ethics, Digital law

## Abstract

Digital phenotyping represents an avenue of consideration in patients' self-management. This scoping review aims to explore the trends in the body of literature on ethical, legal, and social challenges relevant to the implementation of digital phenotyping technologies in healthcare. The study followed the PRISMA-ScR methodology (Tricco et al. in Ann Int Med 169(7):467–473, 2018. https://doi.org/10.7326/M18-0850). The review systematically identified relevant literature, characterised the discussed technology, explored its impacts and the proposed solutions to identified challenges. Overall, the literature, perhaps unsurprisingly, concentrates on technical rather than ethical, legal, and social perspectives, which limits understanding of the more complex cultural and social factors in which digital phenotyping technologies are embedded. ELS issues mostly concern privacy, security, consent, lack of regulation, and issues of adoptability, and seldom expand to more complex ethical issues. Trust was chosen as an umbrella theme of a continuum of major ELS and technical issues. Sustained critical analysis of digital phenotyping showed to be sparse and geographically exclusive. There is a continuum and overlap between ELS issues, suggesting the need for a holistic, interdisciplinary approach to each of the challenges posed by the various technologies of digital phenotyping.

**Supplementary Information:**

The online version contains supplementary material available at 10.1007/s11948-021-00354-1.

## Introduction

### Rationale

Many pressures weigh on our healthcare system, especially in times of pandemic: demographic pressure and rising healthcare costs that test the limits of the solidarity in our healthcare financing system, consumer pressure from users who expect immediate services, and finally, pressure from the professional world amid change with the advent of the challenges of "Industry 4.0". Whether academic, institutional, or commercial, the discourse on global e-health has an optimistic, even anticipatory tone, where connectivity makes it possible to reach hitherto inaccessible regions, to inform populations lacking medical expertise, and to offer medical care at a lower cost. Digital phenotyping has the potential to greatly contribute to these goals. As defined by Torous et al. (2016) and Onnela and Rauch (2016), digital phenotyping is an emerging field that combines research and technology to analyse people's interactions with the digital world and assess users' health and well-being, monitor the effectiveness of treatments, identify psychiatric diseases, predict patient outcomes, support drug trials, and many other applications. Indeed, smartphones and other digital devices allow users to access an almost limitless universe of data and information on every conceivable subject. However, it is important to maintain legislative and regulatory frameworks that preserve rights and freedoms and commonly shared fundamental values, while the equation between connectivity, efficiency, and cost control is not self-evident and deserves to be problematized. The forces at play are economic, political, and commercial—if there is one unique feature of that booming industry, it is the heterogeneity of sectors and interests involved.

Viewed in this light, the progress made in the field of digital technology over the last few decades has given rise to a social revolution we might not yet be ready for. New knowledge in the field of medicine and health is indeed particularly problematic in that it poses difficult and challenging problems, as it questions deeply ingrained social values. Accordingly, questions surrounding bioethics, but also social and legal issues regarding digital phenotyping, have been regularly raised in recent years. These concerns relate to fundamental issues about the values associated with human beings and morals, such as respect for human dignity, solidarity, and privacy. The very nature of patient-physician confidentiality is at stake as well, as we search for a balance between the imperatives of the community and compliance with the rights of the individual. As new technologies proliferate, a growing number of circumstances arise in which it becomes increasingly difficult to reach strategic goals in the management of medical information. These include the management of patient information and documents; coordination of all internal information flows; reliability and security of the system; hosting and storage of data; increased availability; flexible implementation; immediate access to application tools, etc.

Evidently, digital phenotyping and its ramifications raise many questions which require us to rethink the major principles of law and ethics and the legal frameworks in which the rights and systems specific to health care are embedded. The issues at stake also require that all health actors (health authorities, health professionals and institutions, manufacturers, laboratories, patient associations, etc.) be involved in the reflection process and that the viewpoints of other researchers (medicine, philosophy, computer sciences, sociology, psychology, etc.) be integrated into it to understand, in a concerted manner, the adjustments or changes to existing legal frameworks, the limits that must not be crossed, and the epistemological barriers that must be overcome. The following scoping review of a cross-disciplinary body of empirical and theoretical literature will provide an analysis of current trends, their relevance and limitations will give an overview of identified ethical, social, and legal issues relating to the implementation and use of digital phenotyping and will underscore gaps to be further explored. We begin by outlining the methodology of our review. Secondly, we present the results of this review through a critical analysis of the identified texts. Finally, the discussion proposes an in-depth reflection on the main aspects of these trends, highlighting the questions raised by the cross-disciplinary literature review and the research perspectives that aim to answer them. In conclusion, we indicate some avenues for future research. Thus, the present scoping review speaks to an intended audience of bioethicists, political philosophers, researchers in health sciences, computer sciences, media sciences, and all scholars interested in the ethics, regulatory, and policy issues of digital phenotyping technologies.

### Objectives

The objective of this comprehensive scoping review is to assess and identify the state of the art in the scientific production that concerns the ethical, legal and social (ELS) challenges of digital phenotyping, to evaluate its relevance, and locate the gaps in research trends. As a scoping review, the purpose of this study is to aggregate the findings and present an overview of the research rather than to evaluate the quality of the individual studies. The quantitative findings relating to our assessment of the research body will therefore serve to support a narrative commentary regarding our findings. We will follow the PRISMA-ScR framework (Tricco et al., 2018) to categorise and synthesise the literature.

This work could serve as a reference framework for the evaluation of future implementations of digital phenotyping. In doing so, this paper will consider the following critical questions:

- What are the major themes, concepts, and topics discussed in papers about ethical, legal, and social issues (ELSI) of digital phenotyping?
- What are some ethical, social, and legal implications of digital phenotyping today, and what is anticipated for the future?
- What does the literature say about managing the ethics of digital phenotyping?
- What recommendations can we draw from the literature in assessing the ethical, legal, and social implications of the use and implementation of digital phenotyping? And finally,
- What are the gaps in the research and literature about the ELSI of digital phenotyping?

## Methods

### Protocol

**Fig. 1:**
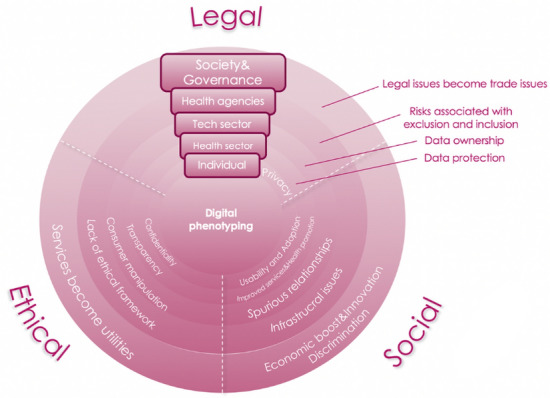
Authors' preliminary thematic scheme related to the ethical, legal, and social issues in the literature about digital phenotyping

The scoping review protocol of this study was drafted based on the PRISMA extension for scoping reviews (PRISMA-ScR) (Tricco et al., 2018). The research team consisted of a bioethicist, a sociologist, and a social anthropologist with expertise in the metatheoretical analysis of the literature. After a pilot screening to establish themes and possible difficulties (see Fig. 1), the research team defined the keywords for the database search of a body of literature on the ELS issues of digital phenotyping. Then, the research team discussed and determined inclusion and exclusion criteria for selected publications, and finally proceeded to the search and analysis.

### Eligibility Criteria

Publications explicitly focused on robotics, big data, AI were excluded if *digital phenotyping* was not mentioned alongside these technologies. We also excluded papers focusing on technical issues without discussing ELS issues. Conference abstracts, scoping and systematic literature reviews were excluded as well.

To be included in the review, publications needed to contain any kind of ethical, social, or legal evaluation or assessment about digital phenotyping and focus on digital phenotyping use in diagnostics and decision-making, both in the medical or anthropotechnic sense. Based on our preliminary search of the literature, we decided to expand the WHO's definition of health (1946). Indeed, consumers, researchers, and other stakeholders may have different understandings of health. Moreover, they may assimilate all digital phenotype-related data to health-related data. We therefore decided to adopt a broader meaning of health, encompassing both wellness and fitness, and referring to a holistic notion of healthcare based on physical, mental, spiritual, emotional and social wellbeing, which are all aspects of a symptomatology from patients' daily lives that digital phenotyping promises to obtain.

Papers were included if they were published in peer-reviewed journals or peer-reviewed books and book chapters written in English Conference proceedings, editorials, bulletins, commentaries, business reports, and dissertations were included as grey literature. As the concept of digital phenotyping was coined in 2016, all publications published after January 2016 and before January 2021 which fit the above criteria were retrieved.

### Information Sources and Search Criteria

In determining our string of keywords, and with the crossdisciplinary objectives of the review in mind, we took into consideration *digital phenotyping* keyword variations across disciplines. We considered common synonyms but also various terms (metonyms, synecdoches, or metaphors) that are associated and reflect a particular understanding of the concept of *digital phenotyping*. Based on our preliminary literature search, and in an effort to use a uniform search string across all databases, we limited the number of keywords relating to *digital phenotyping* to 8, as one of the databases (IEEE Xplore) is limited to 11 keywords, and the 3 remaining keywords were dedicated to ELS issues: *ethical, legal and social*. As search functionalities varied regarding the combination of titles and abstracts, we also limited our search to *titles only* across all databases, which is accepted as a valid search strategy in the literature (Mateen et al., 2013; Rathbone et al., 2017). And finally, English was set as a filter when it was not a default search filter.

To identify relevant publications, we searched the following online bibliographic databases on January 10th, 2021, setting the time frame from January 2016 to December 2020: *PubMed, PsycInfo, Web of Science, Scopus, Ovid,* and *IEEE Xplore*. Each database was searched through their search engine interface using the following keywords: "*ethic*", "legal", "social", "digital phenotyp*", "passive data", "self-tracking", "biometr*", "crowdsensing", "biofeedback", "quantified self", "wearable*". We then proceeded to forward and backward snowballing search from every retrieved publication. To complete our corpus, we proceeded to a search on Google Scholar—as Google's search engine has no standard principles of Boolean operators, we used the keywords "digital phenotype" and "ethical", "social", "legal". We screened the first 10 pages of results and included the publications which did not come out through database search nor snowballing procedure. We subsequently limited our scope to the field of health and well-being through title and abstract screening, followed by full-text screening. Results were retrieved using the export function of each database in the corresponding format. To eliminate duplicates, we used the reference management software Zotero (2016) and for both phases of publication screening (title and abstract and full-text screening), we used the online and/or mobile systematic review software Rayyan (2016).

We identified both theoretical and empirical studies from different disciplines in the health sciences, engineering and technology sciences, the social sciences and humanities: sociology, anthropology, psychology, and social psychology, media and communication sciences, philosophy, as well as art and design. In the same scope, we identified studies that reflect on the ELS issues of digital phenotyping both quantitative and qualitative, and articles explicitly dealing with both the technical aspects (security, practical use, and data outcomes) and ELS issues of said technology. Articles addressing technical issues only, which did not identify ELS issues, have been excluded.

### Selection of Sources of Evidence

In the subsequent phases of the screening process, researchers have worked independently to avoid bias in the review results. Two researchers (AT, AM) have screened all papers independently (double-blind screening of titles and abstracts, followed by a detailed analysis of full-texts), and selected publications according to the set inclusion and exclusion criteria. When we were not able to discern the above information from the title or abstract, the paper was included for further study. The two readers (AT, AM) discussed indicator labelling weekly to avoid the coder's drift and resolved any disagreement of screening decisions with the third reader (AČ).

### Data Charting Process and Data Items

Informed through the heuristic evaluation of preliminary literature search and by the approach taken by prior literature reviews (Cooper & Yon, 2019; Hofmann, 2013; Maher et al., 2019), a meta-data table listing all selected relevant indicators was developed jointly by the authors to determine which variables to extract from our corpus. The first author charted the data which the second author reviewed subsequently. Minor discrepancies were discussed and resolved by all three authors. Some codes were identified ahead of the screening but additional codes were derived through an iterative charting process. The consolidated meta-data table is available in "Annex 1".

From each publication following the first screening, we abstracted the following data and meta-data on article characteristics (e.g., source type, year, first author's country of affiliation, etc.), publication design (e.g., theoretical or empirical), disciplinary background, discussed technology, beneficiaries, etc., and ELS issues mentioned (main challenges identified and classified as ethical, social, or legal).

To categorize the papers by indicators (labels), the following data and meta-data items were extracted from each publication during the full-text screening process. Below is a more detailed description of extracted data and meta-data items:

### Synthesis of Results

We took note of all the publications that met our inclusion criteria and the reasons for excluding certain papers. We analysed the overall results after the full-text screening to present an overview of existing literature on the ELS issues in the field of digital phenotyping. We focused on literature presenting original research and theoretical analyses to identify the breadth (fields of application, users, identified beneficiaries, usability, participant characteristics, etc.) and depth (ELS issues, ex-ante and ex-post approaches) of existing research. The characteristics of the publication included in our corpus are presented in a tabular form in the below sections of this scoping review. We also computed and graphed summaries of charted data frequencies. Finally, we collated, summarized, and reported the findings of our scoping review relating to each of our research questions.

## Results

### Sources of Evidence

Of the 527 articles retrieved from our database search based on title relevance, 88 publications were considered pertinent based on title and abstract screening. After expanding our corpus through forward and backward snowballing, and a Google Scholar search, and after deletion of duplicates (if the same paper was identified in multiple databases), a total of 151 publications met our inclusion criteria.

Both the title and abstract screening and the full-text screening reached a very low conflict percentage (1.8% and 2.6% respectively, as computed by the software Rayyan). Any doubt about the selection of an article was cleared by a third opinion and the decision was taken by consensus. If the full-text article was not available, the authors were contacted by e-mail. The articles were also searched on search websites such as Google Scholar, Academia.edu, ResearchGate. Unavailable full-texts were excluded.

**Fig. 2:**
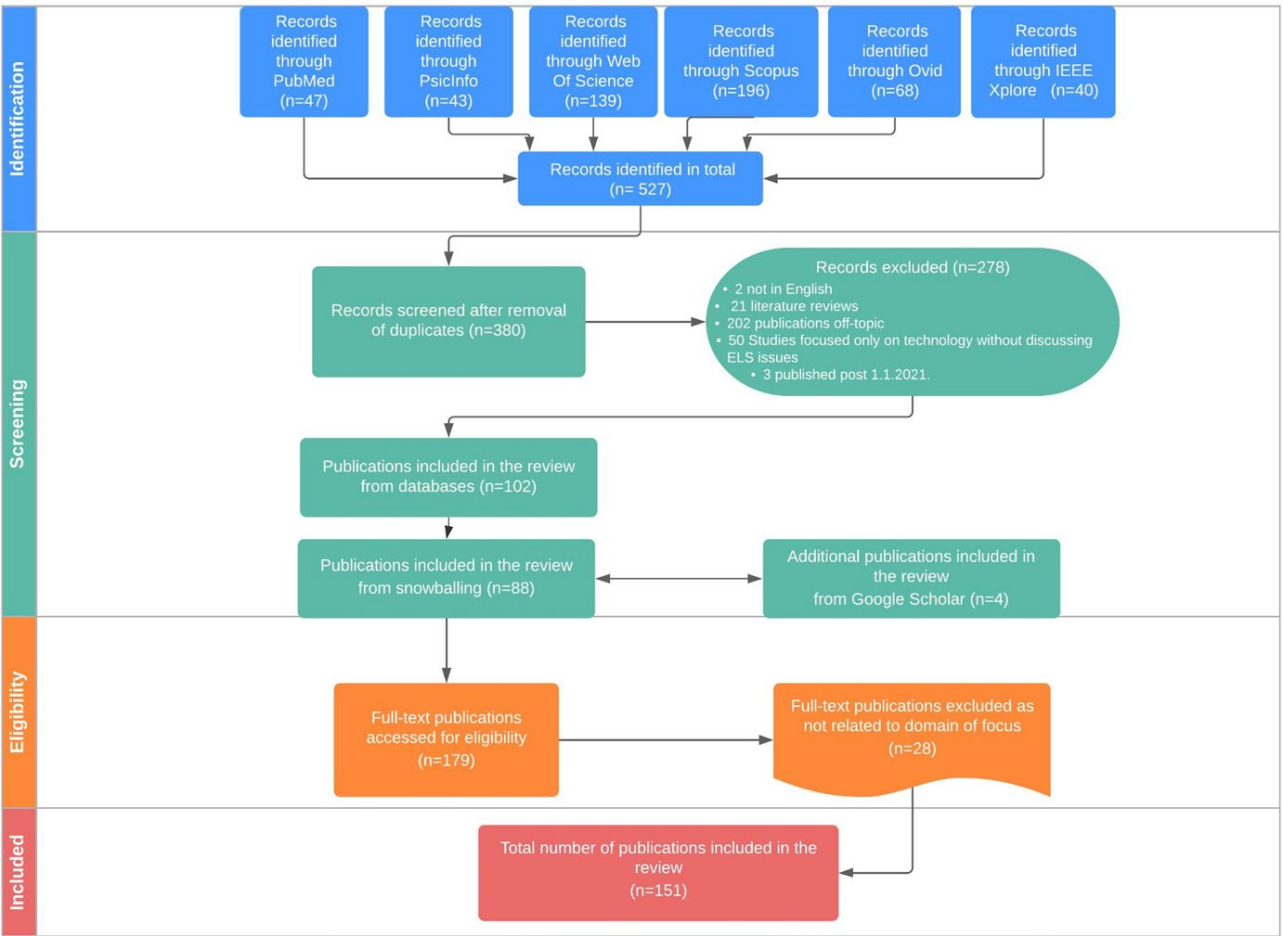
Source selection process from literature databases represented in a flow diagram

Figure 2 below shows the different stages of our search process and the results obtained by stage. The stages are summarized in four phases, namely identification, selection, eligibility (these two intermediate stages consist of excluding articles that do not meet the search criteria), and finally the inclusion phase.

### Results of Individual Sources of Evidence

All indicators in our corpus have been clustered and categorized for heuristic purposes as ethical, legal, social, or technical issues. In Fig. 10, we present the most frequently encountered indicators as well as their source publications. We present below the most relevant outcomes data for our body of selected literature. Full results, graphs, and tables can be accessed in "Annex 1".

**Fig. 3:**
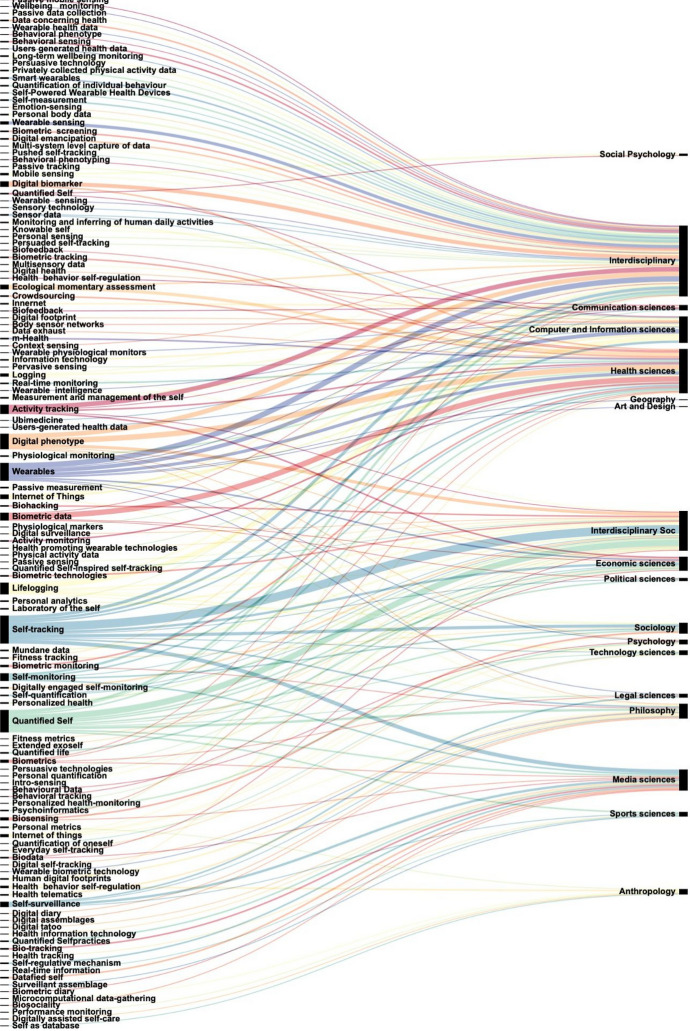
Lexical field of Digital Phenotyping by disciplinary field

While we noted 207 different terms for devices used in the field of digital phenotyping (see "Annex 1"), the most common terms relating to *Digital Phenotyping* referred to in our corpus were: Quantified self; Digital Phenotype; Self-tracking; Wearables; Biometric data; Digital Biomarkers; Internet of Things; Lifelogging; Activity tracking; and Self-surveillance; a series of terms such as Ubimedicine; Reality mining; Psychoinformatics; Innernet were used seldom. The lexical field of Digital Phenotyping (Fig. 3) shows to be inconsistent in its use in the literature.

**Fig. 4:**
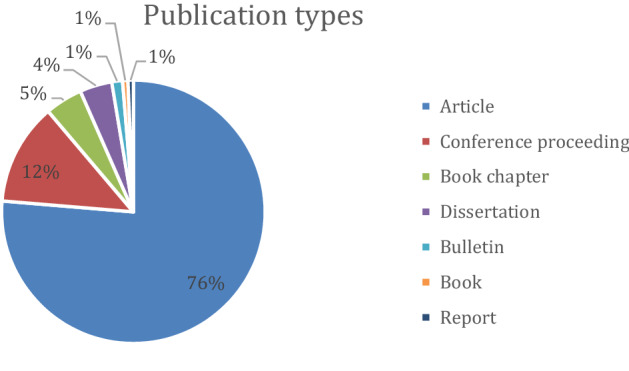
Distribution of the 151 publications

**Fig. 5:**
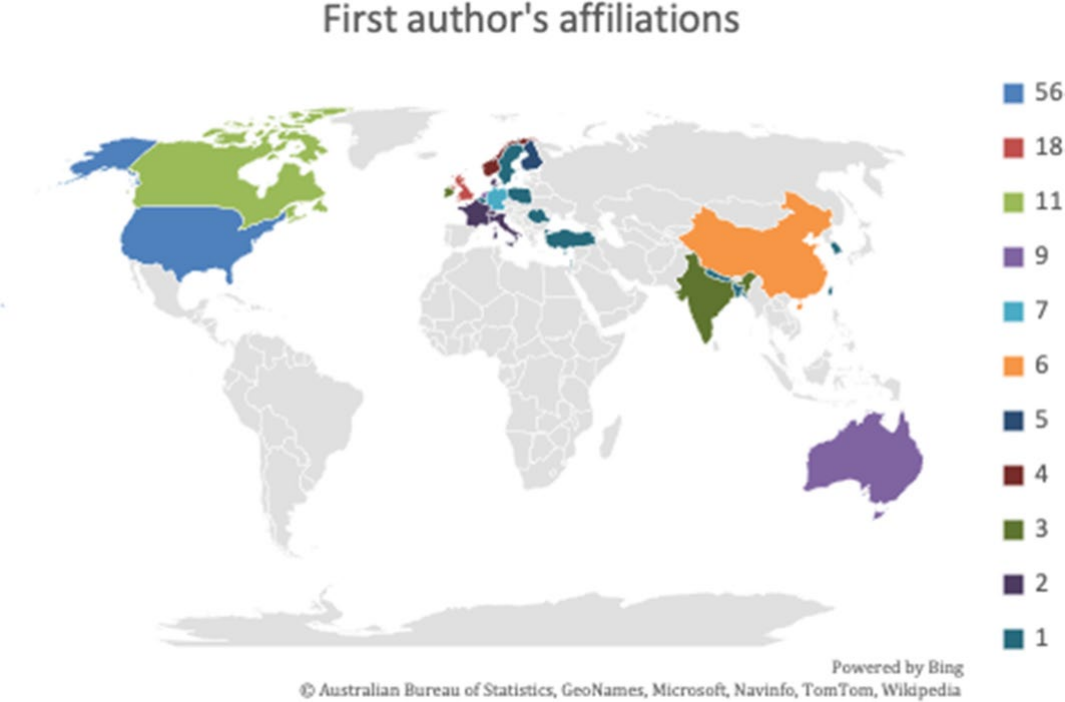
First author's countries of affiliation

**Fig. 6:**
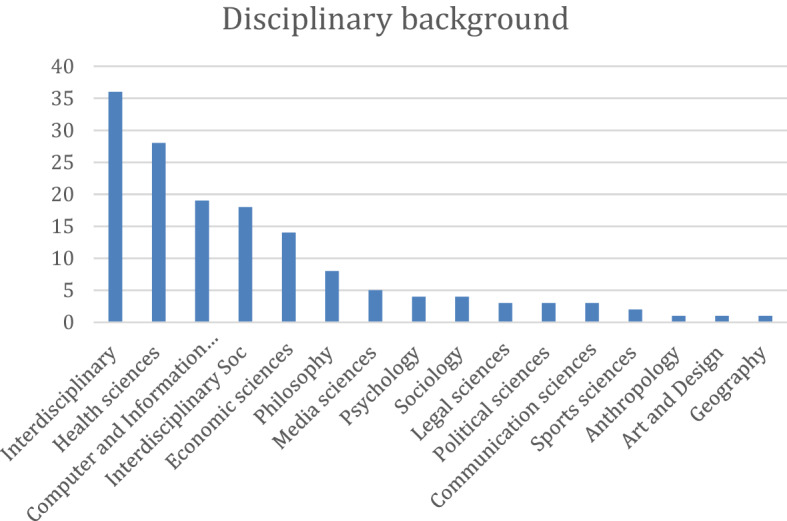
Authors' disciplinary background—Interdisciplinary, health, and computer sciences lead in the scientific production of literature on the ELS issues of digital phenotyping

Out of the 151 publications in our corpus (Fig. 4), 56 were from authors affiliated to a university of the United States (Fig. 5). Amongst the most numerous publications were papers from the United Kingdom (18), Canada (11), and Australia (9); 9 publications originated from the Netherlands, 7 from Germany, 6 from China, 5 from Finland, 4 from Norway, 3 from India, 3 from Ireland, and 2 from respectively France, Italy and Switzerland. Each of the following countries contributed to our corpus with a single publication: Bangladesh, Belgium, Cyprus, Israel, Nepal, Poland, Romania, Singapore, South Korea, Sweden, Taiwan, and Turkey (Fig. 6).

**Fig. 7:**
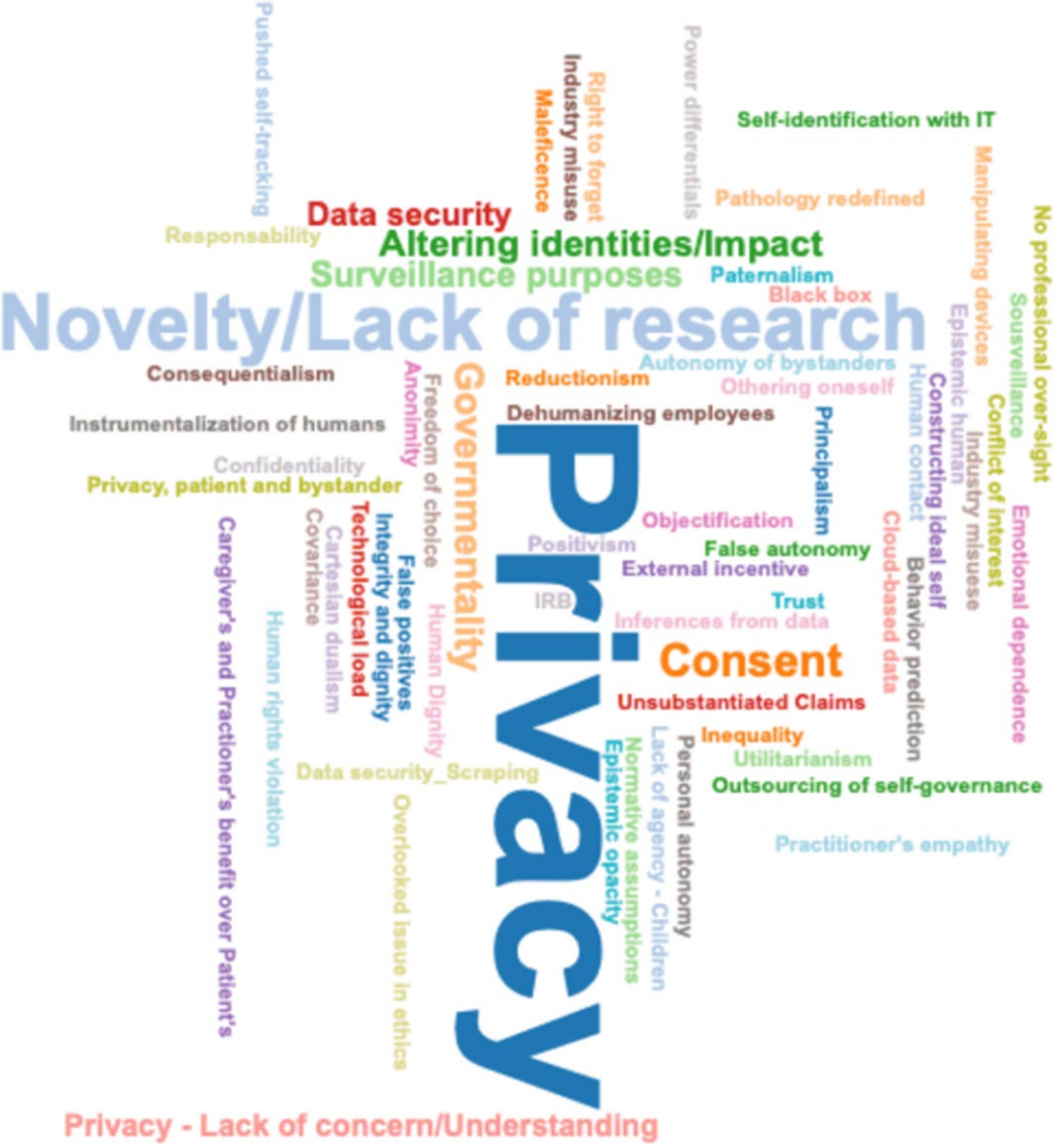
Word cloud of identified *ethical issues*

As seen in Fig. 7, the main ethical challenges identified in the analysis are: Privacy; Lack of research; Consent; Impact on human psychology; Surveillance and Data Security; followed by the Lack of privacy concern; Governmentality; and "Pushed phenotyping" (external incentive). Seldom mentioned ethical concerns are: The right to forget; Utilitarian morality; Reductionism; Lack of professional oversight; Covariance; Technological load; Outsourcing of self-governance; Dual-use technology, etc.

**Fig. 8:**
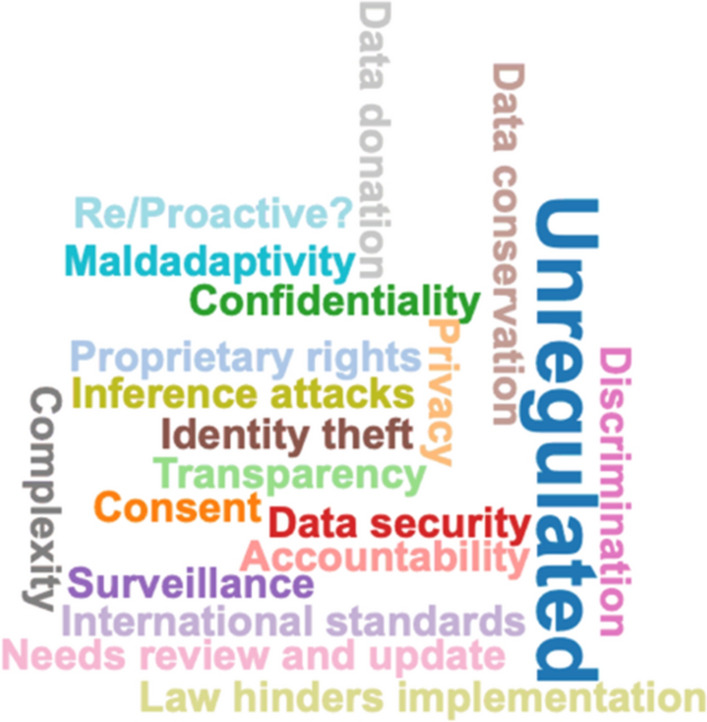
Word cloud of identified *legal issues*

The main legal challenges (Fig. 8) identified in the analysis are: Lack of regulation; Data ownership; Consent; Privacy; Transparency; Data security; Confidentiality; and International legal standards. Seldom mentioned legal concerns are: Legal obstacles to implementation; Inference attacks; Data donation; Identity theft, etc.

**Fig. 9:**
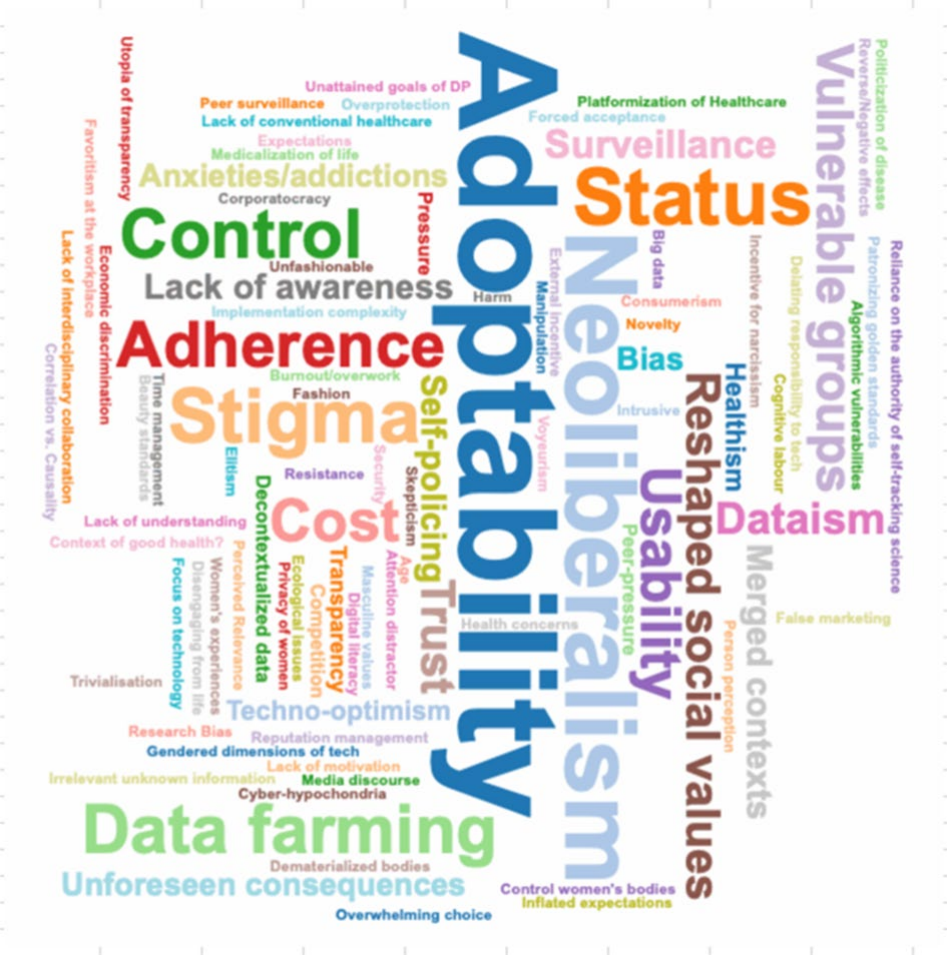
Word cloud of identified *social issues*

**Fig. 10:**
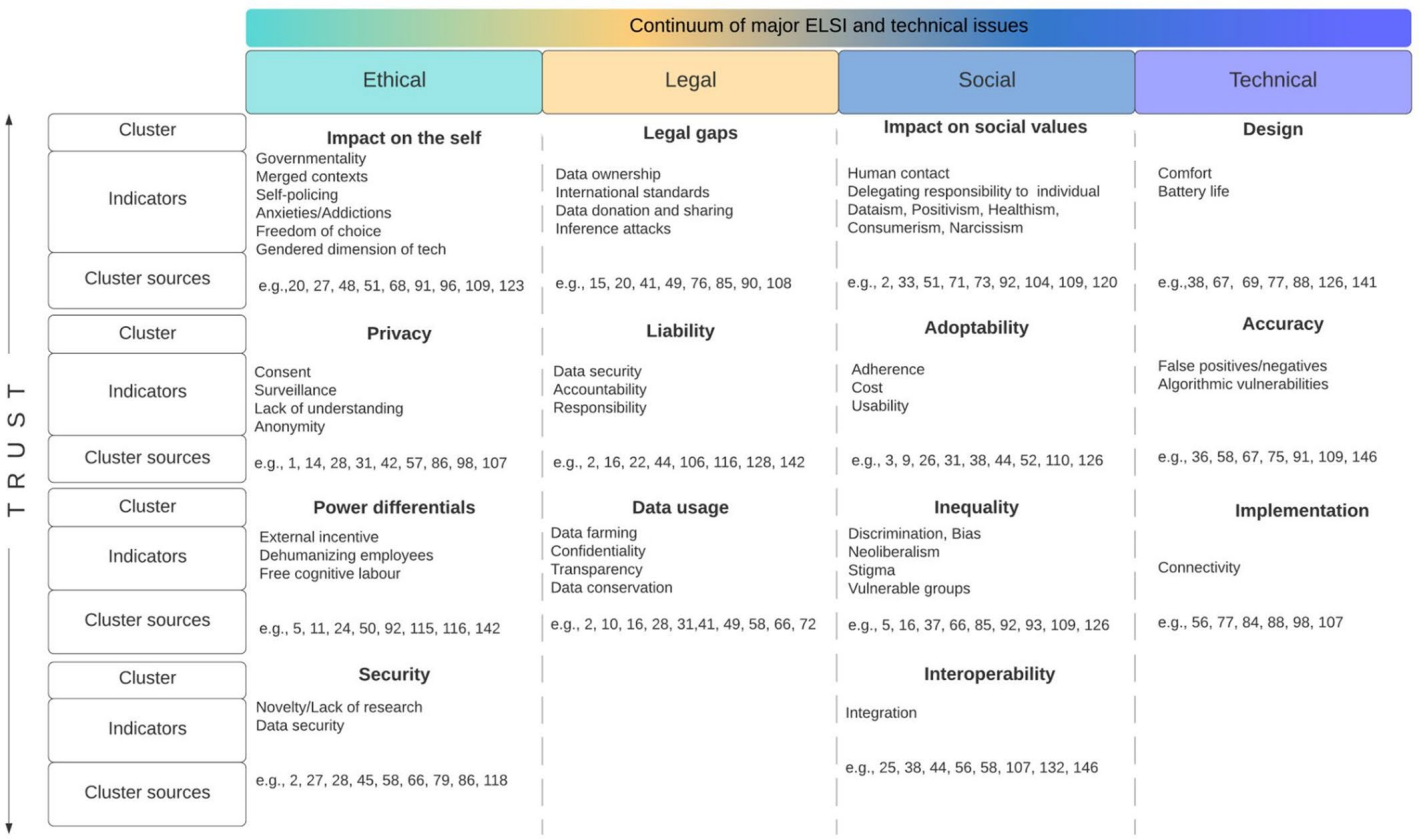
Clusters of identified ELS and technical issues with sources

The main social challenges (Fig. 9) identified in the analysis are: Adoptability/Acceptability; Security and Risk of Harm; Inequality; Neoliberalism; Status-related use of wearables; Stigma; Control; Data farming; Adherence; Cost; Discrimination; Usability; Risk posed to vulnerable groups; Delegating responsibility to individual level; Reshaped social values; Trust; Dataism; Rise of associated costs (insurance); Surveillance; Lack of awareness of any ELS issues; Merged contexts; and Self-policing. Seldom mentioned social concerns are: Voyeurism; Trivialisation; Women's privacy; Lack of conventional healthcare; Inflated expectations; Incentive for narcissism; Cyber-hypochondria; Utopia of transparency; Media discourse; Implementation complexity; Ecological issues, etc. (Fig. 10).

### Synthesis of Results

Following the analysis of the included literature, we identified clusters of ethical, legal, and social concerns voiced by the authors. We have chosen *Trust* as an umbrella term that spans across all ethical, legal, social, and technical challenges—from trusting the design and connectivity of a wearable device to trusting its impact on the self or on social values. As Trust “operates in terms of dispositions, beliefs or cognitions and feelings or affects and emotion [and is] essentially dyadic, between two individuals, even if one of those ‘individuals’ is a collective entity” (Barbalet, 2019), it is essential that the ELS issues of digital phenotyping be addressed in terms of trust building—or “the experiencing of reality that provides `good reasons'” (Möllering, 2001), if the full potential of digital phenotyping is to be realized.

Much of the scientific literature on digital phenotyping is marked by high hopes for the advent of digital technologies to improve mental and physical health and the overall well-being of individuals. The dominant movement of that vein is the *Quantified Self* (e.g., 1, 2, 11, 20, 24, 28, 41, 53, 59, 64, 65, 68, 71, 75, 85, 86, 91, 95, 104, 115, 120, 121, 122, 124, 131, 136, 142), fostered by the expansion of applications and tools for tracking activities and lifestyle, as well as allowing the sharing of users' data (e.g., 2, 27, 41, 46, 49, 50, 75, 84, 86, 96, 121, 134). Ultimately, research within this trend focuses on self-improvement through better knowledge of one's physical body and the self via physiologic measurements (e.g., 2, 46, 71, 75, 96, 110, 115). In this stream, quantification provides a way of knowing oneself that is said to be objective (e.g., 31, 45, 46, 51, 66, 78, 90, 100, 105, 118, 124, 142, 146). There are, however, concerns about the practical and material dimensions of these devices. These concerns revolve around the umbrella-theme of *Trust* (e.g., 2, 17, 23, 32, 46, 64, 66, 67, 71, 75, 76, 78, 96, 107, 115, 119) which spans across all ethical, legal, social, and technical challenges such as their cost (e.g., 4, 9, 33, 38, 67, 76, 85, 91, 100, 129), or the rise of associated costs such as insurance (e.g., 2, 16, 41, 46, 49, 50, 53, 60, 75, 96), their accessibility (e.g., 2, 37, 46, 51, 64, 71, 75, 81, 96, 110, 115, 137), adherence to the long-term use or under-use of such technology (e.g., 3, 5, 9, 33, 38, 40, 46, 54, 71, 72, 75, 85, 110, 114, 115, 126, 133), or the reliability of the data (e.g., 2, 21, 28, 33, 38, 46, 47, 67, 71, 75, 77, 90, 96, 129, 131). As never before had so much data been collected, transmitted, and stored about individuals, the confidentiality and security of these data have also been questioned (e.g., 2, 15, 31, 33, 45, 46, 71, 75, 96, 115). Criticism is particularly pronounced in interdisciplinary studies (e.g., 9, 10, 20, 58, 77, 99, 119), health sciences (e.g., 3,7, 33, 35, 40, 65, 90), computer and information sciences (e.g., 30, 32, 42, 57, 60, 63, 79), and with strong arguments from sociology (e.g., 91, 95, 96, 114) and philosophy (e.g., 67, 71, 86, 102, 113, 147). These critiques have arisen in opposition to the promises of technology in terms of benefits and potential from these new devices. The issue of privacy (e.g., 25, 45, 53, 75, 78, 84, 144), surveillance (e.g., 1, 2, 11, 42, 64, 72), benchmarking (e.g., 27, 41, 43, 104), healthism (e.g., 51, 71, 92, 122) and empowerment (e.g., 27, 51, 67, 78) are problematized and questioned in relation to the possible implications and consequences of the use of wearables to promote health or treat illness. A key thread that binds together the works in this stream is the critique of the instrumentalization of the human body and health by farming data (e.g., 2, 72, 76, 78, 87, 123), dehumanizing employees (e.g., 5, 24, 50, 92, 116) or free cognitive labour (e.g., 11, 115, 142, 147). The focus on purely technical aspects of digital health has been identified as being reductive in light of a more complex reality (e.g., 56, 142, 147).

## Discussion

### Summary of Evidence

**Fig. 11:**
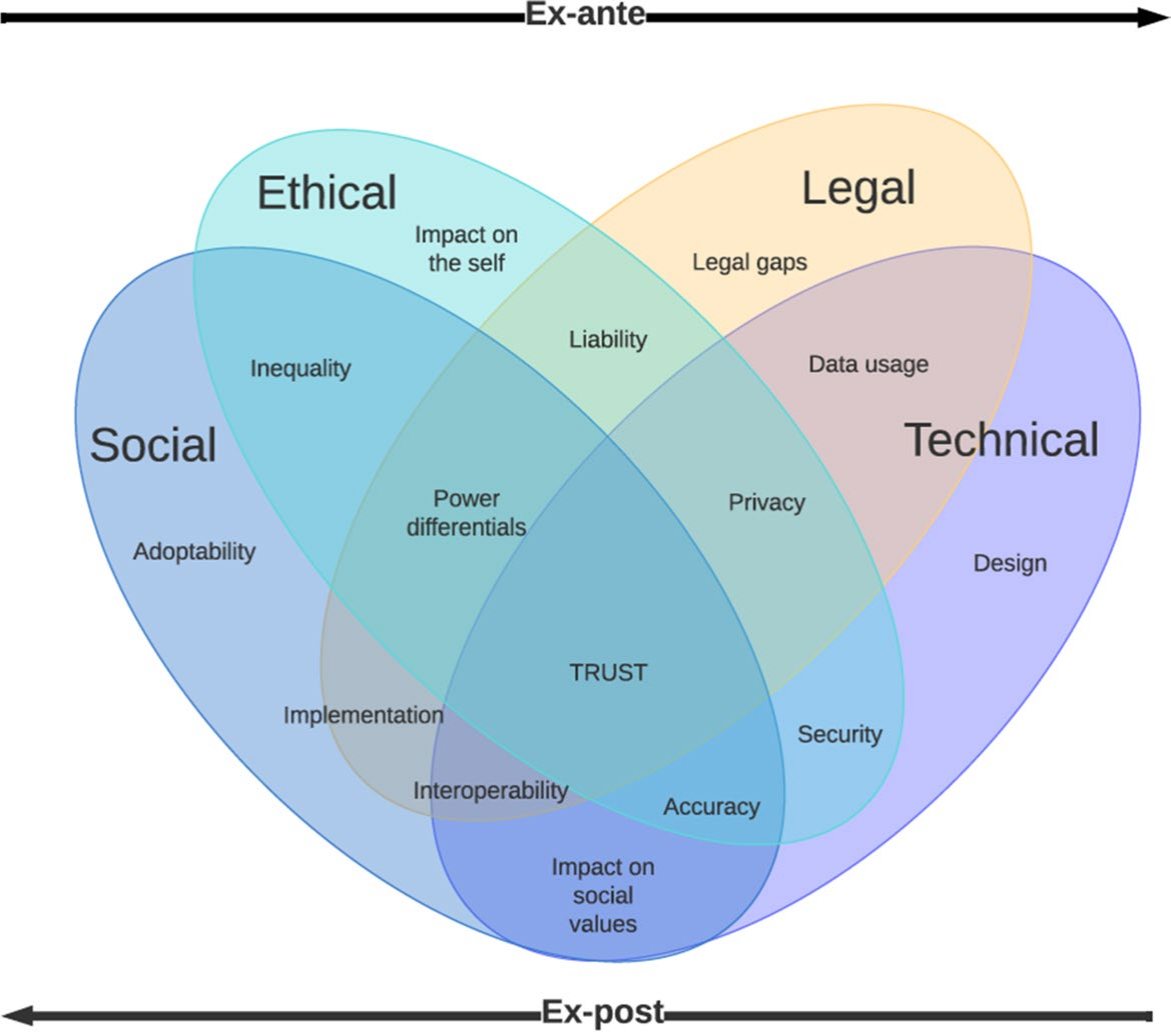
Authors' conceptual scheme representing the ethical, legal, and social issues in the literature about digital phenotyping

This study aimed to explore the major themes, concepts and topics discussed in the literature about ELS issues of digital phenotyping, to uncover the ELS implications of digital phenotyping today and their anticipated future, the various disciplinary approaches and methodologies in assessing the ELS implications of the use and implementation of digital phenotyping, and finally the gaps in the research and literature about the ELS issues of digital phenotyping. A conceptual scheme of identified clusters is represented in Fig. 11.

The detailed examination of the different dimensions that characterize the body of literature on the ELS issues of digital phenotyping suggested several key concerns, particularly concerning the integrity of the digital environment. The degree of *trust* that users attach to access and platform providers in terms of providing secure service environments, as well as the trust placed in governments and regulatory authorities to safeguard the integrity of the digital environment and to prevent adverse effects on individuals and society, is becoming a major parameter affecting the potential of digital phenotyping in the future of healthcare or for its widespread use.

#### Beyond Issues of Consent: Disempowerment

The authors who are critical of digital phenotyping technologies challenge the dominant, often implicit, beliefs that underlie the phenomenon of healthism—for instance, the belief that individuals have at least some control over their lives and can take responsibility for their own health. The notion of empowerment, in relation to one's quality of life, which is widely used in the literature, seems to be closely linked to healthism, which in turn is linked to the issue of self-control. However, these links do not take into account the social and economic determinants of health (e.g., 2, 41, 51, 65, 92, 104). Decontextualized knowledge production and sharing via technologies are seen by some as devaluing the knowledge of practitioners, who bring experience and empathy to medical practices (e.g., 2, 71, 106, 122, 149). Several authors have particularly warned about the need to regulate digital phenotyping and wearables as consumer or medical grade devices (e.g., 2, 17, 23, 30, 106), but only one looked into issues of covariance vs causation (120). The reliability of the data is questioned as well, since devices from the private sector are patented and they are under no legal obligation to disclose the technical characteristics of their algorithms nor their handling of anonymized data (e.g., 26, 50, 78, 91, 100, 109). The critical literature as a whole denounces the reductive nature of the measurement and quantitative evaluation of health (e.g., 2, 51, 85, 87, 104), while a still less prominent trend in the literature considers that the instrumentalization of the human body serves the interests of private companies and not individuals or society as a whole (e.g., 2, 11, 27, 51, 85, 92).

#### The Quantification of Life: Delegating Collective Responsibility to Individuals

Similar questions have been problematized in terms of participatory surveillance (Albrechtslund, 2008; Pagliari & Vijaykumar, 2016), a concept that deals with the quantification of life as a whole (e.g., 65, 72, 75, 86, 91, 98, 123). It is argued that individuals may be taking an active part in diminishing their own power to act through digital technologies, and that connected devices may operate in this way through statutory enticement (e.g., 5, 11, 41, 50, 125, 142). On the other hand, the disconnect between the social and economic dimensions has been problematized through applied research, such as in the treatment of obesity (e.g., 12, 43, 48, 50, 64, 68, 75). Several authors, particularly in philosophy and sociology, point to the reductive biomedical nature of digital health's aspirational approaches. They question the potential benefits of the Quantified Self movement and the technological fix associated with it, especially given that users gradually seem to lose interest in connected devices after only a few months of monitoring (e.g., 3, 9, 26, 85, 97, 105, 114, 133). Consequently, the quantification of the self is generally ad hoc in terms of individual practices and does not extend to medical healthcare practices (e.g., 44, 64, 100, 112). The disincentives, costs, and burdens associated with the introduction of biometric evidence generated by the body and its activity, and concerns about the collection of such data, are significant challenges. However, Sharon (2017) uses an ethnographic approach to show a more nuanced understanding of “data fetishism”, aligned towards “enactments of autonomy, solidarity, and authenticity”. On the other hand, self-monitoring of athletic performance (e.g., 31, 49, 100) would be polarly opposed to this type of practice, given that the competitive incentive to improve is strong and the objectives are more precise among this population. In contrast, individuals in the general population experience a high degree of volatility in their use, given their disengagement from self-knowledge, the body, and health through numbers (e.g., 3, 9, 26, 85, 97, 105, 114, 133). However, only limited research has been conducted on these limitations.

#### Self-Monitoring in the Neoliberal Context: The System at Large as Issue

Even though ELS issues related to digital phenotyping are seldom put in relation to market forces and market power and their mutual effects, the topic of digital health has become the subject of sociological and philosophical reflections inspired by the work of Foucault on biopower transposed to contemporary post-industrial societies (e.g., 5, 20, 27, 48, 65, 85, 116, 125, 140). It criticizes today's tendency of using wearable devices to impact behavior. Health behaviors, understood as practices of the self, reflect the political influences of biopower in terms of disciplining the individual and controlling populations. In this vein, Lupton (2016) highlights the lack of critical distance in research from how digital technologies are used in health. She argues that the prevailing enthusiasm for digital health care is an impediment to critical questioning of the social, cultural, ethical, political, and economic dimensions at stake with these rapid technological developments. Lupton maintains there are deep implications of the focus on digitalization in healthcare, as it leads to new ways of tracking and monitoring the human body, along with increasingly widespread sharing of related data. A central implication of self-quantification is the significant narrowing of accepted social representations of health, wellbeing, and disease (e.g., 71, 92, 121, 122, 124). Biometrics and related measurements, together with comparisons to virtual norms defined by technology proponents and technology-oriented authors, are gradually reducing the range of experiences and ways of articulating the lived experience of individuals and entire populations. A critical approach to digital health thus requires an analysis of how the boundaries between the domains of health, well-being, and illness are influenced by technology, as well as of issues of intimacy, sharing and privacy, and the relationship to the body. In this perspective, embedding the issue of politics is inseparable from the uses of connected objects and the meaning and subjectivity of the users that incorporate them in their daily lives.

#### New standards and Reshaped Social Values

In conjunction with the issue of privacy and surveillance, a distinctly critical trend towards digital health has emerged. It highlights the individualistic and normative character of self-monitoring seen as a means to attain a particular objective. It is argued that each individual is deemed to bear sole responsibility for his or her own health, to the detriment of a more community-based and global approach to healthcare, viewed from a broader public perspective. As Ajana (2018) underscored, “Privacy is increasingly framed as a normative individualistic concept that is inherently in opposition to the collective good”. Various authors denounce the managerial dimension that the use of connected objects brings to health, defined as a purely individual resource (e.g., 87, 109, 122, 123, 131). Authors have discussed self-quantification in terms of the emergence of new reference values through competition and comparison, in conjunction with the production of biometric data, whereby the norm is algorithmically defined (e.g., 27, 41, 43, 86, 97). The trend is that the benchmarks that will determine good health via connected devices will be defined by a phenomenon of healthism or dataism—a notion that places the maintenance of good health, performance, and efficiency above other aspects of life and daily activities (e.g., 51, 64, 71, 87, 92, 121, 122). Particularly skeptical authors claim that such healthism will result in a complete disempowerment, as quantifying the self may well entail the establishment of a homogenous lifestyle characterized by the same patterns of consumption, behaviors, and concerns (e.g., 51, 124). In addition, there are concerns about the relationship to the data produced and collected, the meaning attributed to it, and how this data is transformed into action on the self (Lupton, 2016).

#### Research Gaps and Methodological Issues

Finally, we identified a series of research gaps and methodological issues that must be addressed in future research. First and foremost, this review revealed a problematic use of terminology and a high degree of semantic confusion used in the current scientific literature (Fig. 3). This phenomenon reflects the rapid and constant development of the technology itself, along with how it is marketed, often involving semantic shifts that are difficult to pinpoint. The observed ambiguity of the concept seems to be linked to the ongoing race to produce increasingly effective connected devices, in close connection with the economic interests of public health. Scholars might need to coin new terms which encompass these emerging meanings and possibilities, as well as the critique or connotation they wish to enclose in the terminology. Some will rapidly gain currency (*digital phenotyping*, for instance). However, this hinders cross-disciplinary exchange, while the general public, i.e. the users as well as the data provider of all research, might feel overwhelmed by the complex terminology that emerges from various fields: *digital phenotyping*, *ubimedicine*, or *innernet* are not self-explanatory. We recommend that scholars who target an audience outside of their disciplinary fields adopt clear standard English descriptors such as "self-tracking" or "personal sensing" (Mohr et al., 2020). Moreover, when referring to a wearable device by its trade name, scholarly production can even act as an advertising platform for the most common brands of wearable devices, the brand name of the undisputed market leader in wearable fitness being the most used term for wearable devices (e.g., 28, 41, 43, 53, 72, 115). Furthermore, technological advances in the field are based on representations of what is considered to be healthy in relation to different daily activities (e.g. eating, sleeping, being active), but whose referents are only rarely made explicit. The study of digital phenotyping and wearable devices has resulted in a great deal of confusion in the scientific literature regarding wellness and health, but also in the labelling of the quantification of human activity. We consider it necessary to deconstruct these presuppositions and to analyse the terminology in order to identify rigorous concepts and to delimit the fields of application of the various devices. One way to improve this understanding would be to create an interdisciplinary glossary to mitigate misunderstandings without requiring scholars from a particular discipline to immerse themselves over lengthy periods of time in the literature of another discipline. We thus note the need for a better definition of the terms in use, with a view to a better understanding of the issues at stake, whether between the various stakeholders in the health sector or within a single discipline. Furthermore, conferences tagged as interdisciplinary often remain multidisciplinary, as interdisciplinarity faces intellectual and organizational difficulties (Glasberg, 1997), and such a glossary would help rethinking the directions in international and interdisciplinary conferences which remain essential to the challenge of improving communication and collaboration among the various disciplines and sectors in the field.

Furthermore, the reviewed body of literature highlighted the importance of questioning the boundaries between the notions of health and disease. A widely shared conception of the human body withstands throughout the literature: it can be measured, adjusted, programmed, or controlled by technologies. In other terms, the majority of authors discussing ELS issues of digital phenotyping position themselves as technophiles or technophobes, ranging from the idealization of connected objects with some concern about privacy to strong skepticism about them. This recurrent opposition conceals a widely shared conception of the human body, a common apprehension of a human subject who could externalize and delegate their lived sensations to machines. The body, health, and illness are apprehended as biological facts, with little or no place for such facts as culture and intersubjectivity. Moreover, this dichotomy of positions frequently opposes disciplines: health sciences and to a large extent psychology, of a more positivist and post-positivist persuasion, develop perspectives that have little to do with sociological work or the critical approaches developed on wearable devices, thus resurrecting classic oppositions between positivist and/or post-positivist paradigms, on the one hand, and constructivist and/or subjectivist paradigms, on the other (e.g., 2, 51, 85, 87, 104, 116, 120).

This scoping review highlights the need for longitudinal and contextualized studies in the field of digital health. Despite an unprecedented increase in the use of apps and wearable devices, this has not been the subject of many critical empirical studies: critical socio-cultural or longitudinal psychological analyses are scarce, although it is imperative to study the possible addictive and other psychosocial effects of digital phenotyping related technology, such as orthorexia and its links to healthism and dataism (e.g., 31, 43, 68, 86, 131, 140). Similarly, few interdisciplinary or longitudinal studies have been conducted on the uses of connected devices by various stakeholders, especially the most vulnerable groups (children, the elderly, minorities, people with disabilities, etc.). For instance, as Dow Schüll (2016) reported on her ethnographic fieldwork conducted at the Consumer Electronics Show and its Digital Health Summit, “most of the discussion at the Digital Health Summit, however, focused on the well, not the sick”. The specific know-how and practices developed by individuals or communities using connected objects have also received little attention in the literature outside of the Quantified Self movement or digitally skilled individuals or communities (Sharon, 2017). However, our review testifies to an effort from the part of some researchers to integrate multidisciplinary perspectives in the development of objects and/or interventions in the field of digital health (e.g., 8, 10, 12, 18, 20, 28). In this respect, introducing digital technologies into health systems requires coordination and communication between health practitioners, patients, caregivers, users, engineers, and developers. The authors therefore often advocate for more research and/or a more holistic approach to digital health technologies, which should look into the short term as well as long term benefits or detrimental effect of digital phenotyping, and can take into account the complexity of health practices, as well as the personal rituals and habits of patients or other stakeholders. Several qualitative empirical papers and ethnographies have contributed in that sense (e.g., 3, 5, 11, 16, 25, 31, 56, 70, 89, 97, 122), but we encourage additional qualitative studies looking into the perceptions of all beneficiaries and stakeholders (policymakers, developers, engineers, users, medical practitioners, etc.).

The lack of scientific studies demonstrating the impact of national digital health programs around the globe remains important. We have not found studies discussing the overall cost (legal, infrastructural, human, and environmental) of infrastructure implementation on a social (national) level. The social-ecological impact was overall overlooked in our body of analyzed literature, and only 2 publications questioned the environmental impact of widespread usage of digital phenotyping-related devices (56 and 118). This scoping review also reveals a strong misbalance of scientific production from the Global North and the Global South, the debate on ELS issues being dominated by the Global North, and resulting in cognitive injustice in knowledge production in the realm of digital phenotyping, thus perpetuating the digital divide between North and South. Moreover, usage of mobile technology might differ by gender (Sobieraj & Krämer, 2020). These differences may be explained in part by women's lower financial accessibility to mobile telephony (especially in the world's least developed countries), social norms, fear of harassment, as well as more limited knowledge of digital tools, and should therefore be further evaluated. Gender-focused research will ensure gender equity is adequately investigated and implemented in policies that will apply to self-generated data. An additional issue to that is that many studies are conducted on a small scale, their duration is limited by funding and, possibly, the pressure to publish. Several authors have warned against this tendency (outside the scope of this scoping review as well, see Choat, 2017). This lack of evidence, combined with the lack of cooperation between the private and public sectors, represents a major obstacle to the integration of digital phenotyping into our existing healthcare systems and its interoperability. Adoptability and adherence being a major social issue in the literature, studies in media discourse that analyze the public's social perceptions of ELS issues should be developed. Funding bodies, both governmental and private, can mitigate many of these issues by strategically funding and collating studies that meet the above criteria, ensuring inclusiveness and meaningful contributions that challenge social health inequality on all levels. This approach could address the issue of algorithmic bias as well. Rethinking models of strategic social entrepreneurship, and partnerships with academia, is another avenue to address these issues and create value through corporate social responsibility. Even though scholars must continue examining ELS issues of digital phenotyping from a variety of angles and theoretical positions akin to a certain disciplinary position, only interdisciplinary and intersectoral work can inform a holistic overview of existing issues and therefore produce and operationalize a suitable solution.

### Limitations

To our knowledge, this work represents the first scoping review examining the ethical, legal, and social issues related to digital phenotyping. We recognize however that our defined scope may have excluded important aspects closely related to digital phenotyping such as ELS issues discussed in articles pertaining to digital data generally speaking—intertwined topics of big data, AI, digital technology, etc. However, this scope is in line with our planned research and therefore sufficient for the current study. We hope to develop this review with a broader scope in due course.

Furthermore, we recognize the limitations which pertain to filtering publications in the English language only: English-speaking countries of affiliation may have been considerably less represented had we been able to expand our search to additional world languages. Whether scientific production contributes to English language hegemony, or whether English has become a *lingua franca* for scholars to communicate their findings across the globe is debatable; but although we are aware of this issue, time and resource constraints have had us limit our search for the present study. Additionally, the country of affiliation of the first author is not necessarily the first author's country of activity.

Our keywords search string was limited to 11 keywords across all search databases. We included keywords based on our preliminary scoping of the literature. However, many more terms from the lexical field of digital phenotyping could have been included, such as *lifelogging,* which was “supplanted” by the term *Quantified self*, as claimed by Deborah Lupton (2017, p.1). Furthermore, in an effort to collect a cross-disciplinary body of literature, we decided to include in our search string the keyword *crowdsensing* (Mehdi, 2019; Pryss, 2019), frequently used in Computer and Information sciences—although this keyword resulted in a fairly large volume of publications, they have not been selected passed our title and abstract screening, as these publications did not address ethical, legal nor social issues in the field of digital phenotyping.

Finally, we excluded a fairly large number of publications based on them addressing solely technical issues—a certain number of those papers did look into digital phenotyping in the context of more vulnerable groups such as the elderly, children with autism syndrome, and others. This confirms the above-stated point that most scholarly studies addressing ELS issues of digital phenotyping look into the use of this technology by healthy groups.

### Concluding Remarks

The objective of this scoping review was to collate the ethical, social, and legal concerns and arguments surrounding digital phenotyping in scholarly publications, to identify the potential impacts and consequences of this particular technology, to locate the gaps in the literature, and to orient academic efforts to address the concerns of digital phenotyping as a future solution for present-day challenges. Alongside the manifest issues of security, privacy, or consent, this scoping review presents distinctive ELS issues related to several stakeholders involved with these technologies (i.e., caregivers, physicians, researchers, designers, engineers, researchers, etc.). Some of these ethical issues are relatively under-represented in published scholarly debates. This implies that there are important concerns that have not been fully or sufficiently considered from a holistic perspective. Firstly, given the impact of such research on the lives of individuals and patients, it seems essential to conduct empirical studies through field research on the dynamics of digital phenotyping technology seen as a social artifact; on the psychosociological influences of these technologies on the individual, interpersonal, societal, and global level; and on the definition of the boundaries between well-being and health and illness. In the same vein, the physical and psychological repercussions of wearable devices need to be examined from the point of view of various profiles of users, based on their experience within their specific social and cultural contexts.

Although the most common theoretical approach used in our body of literature were various theories of technology acceptance, another promising avenue in this direction could be explored through supradisciplinary Social representation theory (Moscovici, 2001) and Actor-Network Theory (Law, 2009). Indeed, the existing debates mainly focused on the promises associated with new health technologies, require particular attention to "making the unfamiliar familiar" (Christidou et al., 2004) and to these objects as mediators of meanings from the individual, interpersonal, societal, and legal perspective. Introducing new technological tools into the field of health is bound to be confronted with the modes of reasoning and viewpoints of the various groups and profiles of users for whom it is intended. Considering the many studies that testify to users' lack of concern for issues of privacy (e.g., 17, 42, 49, 51, 96), it seems legitimate to assume that the vast majority of people, ranging from the enthusiasts of the *Quantified Self* movement to the technically unskilled person, do not have the expert technical understanding to foresee neither all the possible benefits nor the issues which might arise from implementing these technologies on a wider societal scale—physical health issues (eyesight, posture, insomnia), psychosocial issues (addictions, orthorexia, harmed relationships, breaches of privacy), cost of infrastructural implementation, etc. Neither does a legal practitioner, for instance, who will rarely have the necessary level of expert knowledge to foresee all the ramifications of incorporating the rapidly expanding field of digital phenotyping into our soon-to-be globalized healthcare systems. Because of this velocity, the regulatory sector seems to be the least productive both in its critique and its reactivity, largely overwhelmed by the inappropriateness of existing laws to address the emerging legal issues linked to digital phenotyping and its constellation of devices. Moreover, the legal sciences were amongst the most scarcely represented disciplines in our corpus while the lack of regulation was cited as the most prominent issue in that sphere. In this respect, interdisciplinary collaboration from all key fields, qualitative methods, and longitudinal studies that allow for the follow-up of users in the context of their daily lives offer significant potential for gaining an all-around better understanding.

On the other hand, as many benefits it could potentially bring, the history of technology shows that a theoretically usable technological innovation may not necessarily result in its actual wider acceptance. The role of scholars is therefore key to establishing what good practices of digital phenotyping are, as a best practice guidance specific to digital phenotyping is currently lacking. Funding bodies can encourage international partnerships at the service or knowledge production, not national interest. The Covid-19 pandemic revealed not only how crucial international cooperation is in the field of healthcare, but also huge socioeconomic and racial inequalities that underpin the quality of received healthcare.

To conclude, an international, intersectoral and interdisciplinary collaboration integrating participatory designs and anticipatory ethics is key to respond to the pressing need for updated regulation of digital technologies in healthcare. However, the field still has to find its own forum for a more fruitful scientific collaboration. So far published literature, even if notably interdisciplinary, uses specialized terminology that hinders interdisciplinary exchanges. The field could benefit from specialized conferences or workshops, special issues in scientific journals, post-disciplinary confrontation of arguments, and customarily publishing scoping and systematic reviews. To achieve true digital emancipation for all social actors while preserving the delicate ecological balance of our environment from the mass production of such technology, we must coalesce all of our assets. Until then, wear with care!

## Supplementary Information

Below is the link to the electronic supplementary material.

Supplementary file1 (XLSX 10579 kb)

Supplementary file2 (XLSX 29 kb)

Supplementary file3 (XLSX 42 kb)

Supplementary file4 (DOCX 33 kb)
