# Supplementary material for: Ethical, Legal and Social Issues of Digital Phenotyping as a Future Solution for Present-Day Challenges: A Scoping Review
Source: Sci Eng Ethics. 2021 Dec 20;28(1):1. doi: 10.1007/s11948-021-00354-1 (PMC8686352; doi:10.1007/s11948-021-00354-1)
Supplement: Supplementary file 4 — Supplementary file4 (DOCX 33 kb) [file 11948_2021_354_MOESM4_ESM.docx]

ANNEX 4: Table of selected publications

| **In-text citation number** | **Selected publications** |
| --- | --- |
| 1 | Brown, N. A., Blake, A. B., & Sherman, R. A. (2017). A snapshot of the life as lived: Wearable cameras in social and personality psychological science. Social Psychological and Personality Science, 8(5), 592-600. |
| 2 | Catoir‐Brisson, M. J. (2017). The Quantified Self and Mobile Health Applications: From Information and Communication Sciences to Social Innovation by Design. Internet of Things: Evolutions and Innovations, 4, 139-168. |
| 3 | Pope, Z. C., Barr-Anderson, D. J., Lewis, B. A., Pereira, M. A., & Gao, Z. (2019). Use of wearable technology and social media to improve physical activity and dietary behaviors among college students: a 12-week randomized pilot study. International journal of environmental research and public health, 16(19), 3579. |
| 4 | Gowin, M., Wilkerson, A., Maness, S., Larson, D. J., Crowson, H. M., Smith, M., & Cheney, M. K. (2019). Wearable activity tracker use in young adults through the lens of social cognitive theory. American Journal of Health Education, 50(1), 40-51. |
| 5 | Maltseva, K. (2020). Wearables in the workplace: The brave new world of employee engagement. Business Horizons, 63(4), 493-505. |
| 6 | Zhu, Y., Dailey, S. L., Kreitzberg, D., & Bernhardt, J. (2017). “Social networkout”: Connecting social features of wearable fitness trackers with physical exercise. Journal of health communication, 22(12), 974-980. |
| 7 | Liao, J., Xiao, H. Y., Li, X. Q., Sun, S. H., Liu, S. X., Yang, Y. J., & Xu, D. R. (2020). A social group-based information-motivation-behavior skill intervention to promote acceptability and adoption of wearable activity trackers among middle-aged and older adults: cluster randomized controlled trial. JMIR mHealth and uHealth, 8(4), e14969. |
| 8 | Liddle, J. J., Burdon, M., Ireland, D., Carter, A., Knuepffer, C., Milevskiy, N., ... & Hall, W. (2016). Balancing self-tracking and surveillance: Legal, ethical and technological issues in using smartphones to monitor communication in people with health conditions. Journal of law and medicine, 24(2), 387-397. |
| 9 | Bagot, K. S., Matthews, S. A., Mason, M., Squeglia, L. M., Fowler, J., Gray, K., ... & Patrick, K. (2018). Current, future and potential use of mobile and wearable technologies and social media data in the ABCD study to increase understanding of contributors to child health. Developmental cognitive neuroscience, 32, 121-129. |
| 10 | Martinez-Martin, N., Insel, T. R., Dagum, P., Greely, H. T., & Cho, M. K. (2018). Data mining for health: staking out the ethical territory of digital phenotyping. NPJ digital medicine, 1(1), 1-5. |
| 11 | Oravec, J. A. (2020). Digital iatrogenesis and workplace marginalization: some ethical issues involving self-tracking medical technologies. Information, Communication & Society, 23(14), 2030-2046. |
| 12 | Régnier, F., & Chauvel, L. (2018). Digital inequalities in the use of self-tracking diet and fitness apps: interview study on the influence of social, economic, and cultural factors. JMIR mHealth and uHealth, 6(4), e9189. |
| 13 | Stanghellini, G., & Leoni, F. (2020). Digital phenotyping: Ethical issues, opportunities, and threats. Frontiers in Psychiatry, 11, 473. |
| 14 | Olimid, A. P., Rogozea, L. M., & Olimid, D. A. (2018). Ethical approach to the genetic, biometric and health data protection and processing in the new EU General Data Protection Regulation (2018). Romanian journal of morphology and embryology= Revue roumaine de morphologie et embryologie, 59(2), 631-636. |
| 15 | Dagum, P., & Montag, C. (2019). Ethical considerations of digital phenotyping from the perspective of a healthcare practitioner. In Digital phenotyping and mobile sensing (pp. 13-28). Springer, Cham. |
| 16 | Anaya, L. S., Alsadoon, A., Costadopoulos, N., & Prasad, P. W. C. (2018). Ethical implications of user perceptions of wearable devices. Science and engineering ethics, 24(1), 1-28. |
| 17 | Chang, V., Xu, X., Wong, B., & Mendez, V. (2019, May). Ethical problems of smart wearable devices. In 4th International Conference on Complexity, Future Information Systems and Risk (pp. 121-129). SciTePress. |
| 18 | Ouverson, K., Kelly, N., & Gilbert, S. B. (2017, July). Fashion and technology: Implications for the social acceptability of a wearable device. In International Conference on Human-Computer Interaction (pp. 203-213). Springer, Cham. |
| 19 | Tang, X., Miller, S. C., & Litzinger, T. A. (2016, May). From nanosystems to ethical eco-systems: designing a workshop for graduate researchers on self-powered wearable health devices. In 2016 IEEE International Symposium on Ethics in Engineering, Science and Technology (ETHICS) (pp. 1-5). IEEE. |
| 20 | Baker, C., & Sicchio, K. (2017, July). Hacking the Body 2.0: ethics in wearable tech, etextiles design and data collection in performance. In International Conference on Human-Computer Interaction (pp. 623-627). Springer, Cham. |
| 21 | Murzyn, K., & Williams, A. D. (2018). Image reprocessing via wearable cameras: effects on memory recall and rumination after a social-stress task. Mhealth, 4. |
| 22 | Yang, S., Gao, B., Jiang, L., Jin, J., Gao, Z., Ma, X., & Woo, W. L. (2018). IoT structured long-term wearable social sensing for mental wellbeing. IEEE Internet of Things Journal, 6(2), 3652-3662. |
| 23 | McCall, I. C., Lau, C., Minielly, N., & Illes, J. (2019). Owning ethical innovation: claims about commercial wearable brain technologies. Neuron, 102(4), 728-731. |
| 24 | Heikkilä, P., Honka, A., Mach, S., Schmalfuß, F., Kaasinen, E., & Väänänen, K. (2018, October). Quantified factory worker-expert evaluation and ethical considerations of wearable self-tracking devices. In Proceedings of the 22nd International Academic Mindtrek Conference (pp. 202-211). |
| 25 | Chang, R. C. S., Lu, H. P., Yang, P., & Luarn, P. (2016). Reciprocal reinforcement between wearable activity trackers and social network services in influencing physical activity behaviors. JMIR mHealth and uHealth, 4(3), e5637. |
| 26 | Gupta, A., Dhiman, N., Yousaf, A., & Arora, N. (2020). Social comparison and continuance intention of smart fitness wearables: an extended expectation confirmation theory perspective. Behaviour & Information Technology, 1-14. |
| 27 | Kent, R. (2018). Social media and self-tracking: Representing the ‘health self’. In Self-tracking (pp. 61-76). Palgrave Macmillan, Cham. |
| 28 | Kreitmair, K. V., & Cho, M. K. (2017). The neuroethical future of wearable and mobile health technology. Neuroethics: Anticipating the future, 80. |
| 29 | Sundrup, R., Windeler, J., & Froehle, C. (2019, June). The Social Implications of Wearable Information Technologies: Extending IT Identity Theory. In Proceedings of the 2019 on Computers and People Research Conference (pp. 103-108). |
| 30 | Kelly, N., & Gilbert, S. B. (2018, September). The wearer, the device, and its use: Advances in understanding the social acceptability of wearables. In Proceedings of the Human Factors and Ergonomics Society Annual Meeting (Vol. 62, No. 1, pp. 1027-1031). Sage CA: Los Angeles, CA: SAGE Publications. |
| 31 | Karkazis, K., & Fishman, J. R. (2017). Tracking US professional athletes: The ethics of biometric technologies. The American Journal of Bioethics, 17(1), 45-60. |
| 32 | Tuovinen, L., & Smeaton, A. F. (2019, June). Unlocking the black box of wearable intelligence: ethical considerations and social impact. In 2019 IEEE Congress on Evolutionary Computation (CEC) (pp. 3235-3243). IEEE. |
| 33 | Fisch, M. J., Chung, A. E., & Accordino, M. K. (2016). Using technology to improve cancer care: social media, wearables, and electronic health records. American Society of Clinical Oncology Educational Book, 36, 200-208. |
| 34 | Jiang, L., Gao, B., Gu, J., Chen, Y., Gao, Z., Ma, X., ... & Woo, W. L. (2018). Wearable long-term social sensing for mental wellbeing. IEEE Sensors Journal, 19(19), 8532-8542. |
| 35 | Dewa, L. H., Lavelle, M., Pickles, K., Kalorkoti, C., Jaques, J., Pappa, S., & Aylin, P. (2019). Young adults’ perceptions of using wearables, social media and other technologies to detect worsening mental health: A qualitative study. PLoS One, 14(9), e0222655. |
| 36 | Montag, C., & Elhai, J. D. (2019). A new agenda for personality psychology in the digital age?. Personality and Individual Differences, 147, 128-134. |
| 37 | Geary-Teeter, A., & Hosseini Ghomi, R. (2020). A Proposed Framework for Addressing Social Justice Concerns in Future Digital Biomarker Research. |
| 38 | O’Sullivan, H. (2019). A Usage and Motivational Model for Wearable Technology: A Users' Perspective (Doctoral dissertation, RMIT University). |
| 39 | Minielly, N., Hrincu, V., & Illes, J. (2020). A view on incidental findings and adverse events associated with neurowearables in the consumer marketplace. In Developments in Neuroethics and Bioethics (Vol. 3, pp. 267-277). Academic Press. |
| 40 | Zhang, Y., Fang, Y., Xu, Y., Xiong, P., Zhang, J., Yang, J., ... & Tan, X. (2020). Adherence with blood pressure monitoring wearable device among the elderly with hypertension: The case of rural China. Brain and behavior, 10(6), e01599. |
| 41 | Ajana, B. (2018). Communal self-tracking: Data philanthropy, solidarity and privacy. In Self-tracking (pp. 125-141). Palgrave Macmillan, Cham. |
| 42 | Aktypi, A., Nurse, J. R., & Goldsmith, M. (2017). Unwinding Ariadne's identity thread: Privacy risks with fitness trackers and online social networks. In Proceedings of the 2017 on Multimedia Privacy and Security (pp. 1-11). |
| 43 | Alter, A. (2017). Irresistible: The rise of addictive technology and the business of keeping us hooked. Penguin. |
| 44 | Sikka, R. S., Baer, M., Raja, A., Stuart, M., & Tompkins, M. (2019). Analytics in sports medicine: implications and responsibilities that accompany the era of big data. JBJS, 101(3), 276-283. |
| 45 | Areán, P. A., Ly, K. H., & Andersson, G. (2016). Mobile technology for mental health assessment. Dialogues in clinical neuroscience, 18(2), 163. |
| 46 | Knowles, B., Smith-Renner, A., Poursabzi-Sangdeh, F., Lu, D., & Alabi, H. (2018). Uncertainty in current and future health wearables. Communications of the ACM, 61(12), 62-67. |
| 47 | Becker, M. (2018). Understanding users’ health information privacy concerns for health wearables. |
| 48 | Bhattacharya, A., Kolovson, S., Sung, Y. C., Eacker, M., Chen, M., Munson, S. A., & Kientz, J. A. (2018). Understanding pivotal experiences in behavior change for the design of technologies for personal wellbeing. Journal of biomedical informatics, 79, 129-142. |
| 49 | Purcell, R. H., & Rommelfanger, K. S. (2017). Biometric Tracking From Professional Athletes to Consumers. The American Journal of Bioethics, 17(1), 72-74. |
| 50 | Challa, N., Yu, S., & Kunchakarra, S. (2017). Wary about wearables: Potential for the exploitation of wearable health technology through employee discrimination and sales to third parties. Intersect: The Stanford Journal of Science, Technology, and Society, 10(3). |
| 51 | Charitsis, V. (2019). Survival of the (data) fit: Self-surveillance, corporate wellness, and the platformization of healthcare. Surveillance & Society, 17(1/2), 139-144. |
| 52 | Choi, J., & Kim, S. (2016). Is the smartwatch an IT product or a fashion product? A study on factors affecting the intention to use smartwatches. Computers in Human Behavior, 63, 777-786. |
| 53 | Christovich, M. M. (2016). Why Should We Care What Fitbit Shares-A Proposed Statutroy Solution to Protect Sensative Personal Fitness Information. Hastings Comm. & Ent. LJ, 38, 91. |
| 54 | Jin, D. (2019). Consumer self-tracking behavior: An investigation of the drivers and outcomes of self-tracking. |
| 55 | Coorevits, L., & Coenen, T. (2016). The rise and fall of wearable fitness trackers. In Academy of Management. |
| 56 | Nayak, B., Bhattacharyya, S. S., & Krishnamoorthy, B. (2019). Democratizing health insurance services; accelerating social inclusion through technology policy of health insurance firms. Business Strategy & Development, 2(3), 242-252. |
| 57 | Koelle, M. (2020). Designing Socially Acceptable Body-worn Cameras. OlWIR, Oldenburger Verlag für Wirtschaft, Informatik und Recht. |
| 58 | Coravos, A., Khozin, S., & Mandl, K. D. (2019). Developing and adopting safe and effective digital biomarkers to improve patient outcomes. NPJ digital medicine, 2(1), 1-5. |
| 59 | Didžiokaitė, G., Saukko, P., & Greiffenhagen, C. (2018). The mundane experience of everyday calorie trackers: Beyond the metaphor of Quantified Self. New Media & Society, 20(4), 1470-1487. |
| 60 | Do, Q., Martini, B., & Choo, K. K. R. (2017). Is the data on your wearable device secure? An Android Wear smartwatch case study. Software: Practice and Experience, 47(3), 391-403. |
| 61 | Li, L., & Peng, W. (2020). Does Health Information Technology Promote Healthy Behaviors? The Mediating Role of Self-Regulation. Health communication, 35(14), 1772-1781. |
| 62 | Gao, Z., & Lee, J. E. (2019). Emerging technology in promoting physical activity and health: Challenges and opportunities. |
| 63 | Wicaksono, A. Envisioning the Future of Healthcare Through Wearable Technology. |
| 64 | Esmonde, K. (2020). ‘There’s only so much data you can handle in your life’: accommodating and resisting self-surveillance in women’s running and fitness tracking practices. Qualitative Research in Sport, Exercise and Health, 12(1), 76-90. |
| 65 | Esmonde, K., & Jette, S. (2020). Assembling the ‘Fitbit subject’: A Foucauldian-sociomaterialist examination of social class, gender and self-surveillance on Fitbit community message boards. Health, 24(3), 299-314. |
| 66 | Jacobson, N. C., Bentley, K. H., Walton, A., Wang, S. B., Fortgang, R. G., Millner, A. J., ... & Coppersmith, D. D. (2020). Ethical dilemmas posed by mobile health and machine learning in psychiatry research. Bulletin of the World Health Organization, 98(4), 270. |
| 67 | Novitzky, P. (2016). Ethics of ambient assisted living technologies for persons with dementia (Doctoral dissertation, Dublin City University). |
| 68 | Etkin, J. (2016). The hidden cost of personal quantification. Journal of consumer research, 42(6), 967-984. |
| 69 | Adapa, A., Nah, F. F. H., Hall, R. H., Siau, K., & Smith, S. N. (2018). Factors influencing the adoption of smart wearable devices. International Journal of Human–Computer Interaction, 34(5), 399-409. |
| 70 | Fuller, D., Shareck, M., & Stanley, K. (2017). Ethical implications of location and accelerometer measurement in health research studies with mobile sensing devices. Social Science & Medicine, 191, 84-88. |
| 71 | Gabriels, K., & Moerenhout, T. (2018). Exploring entertainment medicine and professionalization of self-care: interview study among doctors on the potential effects of digital self-tracking. Journal of medical Internet research, 20(1), e10. |
| 72 | Gilroy-Scott, C. (2017). Surveillance by Fitbit?. Occupational Health & Wellbeing, 69(7), 17. |
| 73 | Henriksen, A., Mikalsen, M. H., Woldaregay, A. Z., Muzny, M., Hartvigsen, G., Hopstock, L. A., & Grimsgaard, S. (2018). Using fitness trackers and smartwatches to measure physical activity in research: analysis of consumer wrist-worn wearables. Journal of medical Internet research, 20(3), e9157. |
| 74 | Homewood, S. (2018, April). Designing for the changing body: A feminist exploration of self-tracking technologies. In Extended Abstracts of the 2018 CHI Conference on Human Factors in Computing Systems (pp. 1-4). |
| 75 | Hoy, M. B. (2016). Personal activity trackers and the quantified self. Medical reference services quarterly, 35(1), 94-100. |
| 76 | Henne, K. (2017). “I Felt Like a Lab Rat”: The Importance of Power and Context in Understanding Biometric Technologies. The American Journal of Bioethics, 17(1), 63-65. |
| 77 | Kinnunen, M., Ervasti, M., Jutila, M., Pantsar, S., Sesay, A. M., Pääkkönen, S., ... & Alasaarela, E. (2016). Improving the well-being and safety of children with sensors and mobile technology. Journal of technology in human services, 34(4), 359-375. |
| 78 | Insel, T. R. (2017). Digital phenotyping: technology for a new science of behavior. Jama, 318(13), 1215-1216. |
| 79 | Baker, S. B., Xiang, W., & Atkinson, I. (2017). Internet of things for smart healthcare: Technologies, challenges, and opportunities. IEEE Access, 5, 26521-26544. |
| 80 | Gagliardi, N. (2019). Is There a Color to Context?: Exploring Domain Associations in Wearable Technology. |
| 81 | Naslund, J. A., Aschbrenner, K. A., & Bartels, S. J. (2016). Wearable devices and smartphones for activity tracking among people with serious mental illness. Mental health and physical activity, 10, 10-17. |
| 82 | Karakaya, M., Bostan, A., & Gökçay, E. (2016). How secure is your smart watch. International Journal of Information Security Science, 5(4), 90-95. |
| 83 | Kelly, N. (2016). The WEAR Scale: Development of a measure of the social acceptability of a wearable device (Doctoral dissertation, Iowa State University). |
| 84 | Kinnunen, M., Mian, S. Q., Oinas-Kukkonen, H., Riekki, J., Jutila, M., Ervasti, M., ... & Alasaarela, E. (2016). Wearable and mobile sensors connected to social media in human well-being applications. Telematics and Informatics, 33(1), 92-101. |
| 85 | Kristensen, D. B., & Ruckenstein, M. (2018). Co-evolving with self-tracking technologies. New Media & Society, 20(10), 3624-3640. |
| 86 | Lanzing, M. (2016). The transparent self. Ethics and Information Technology, 18(1), 9-16. |
| 87 | Leaver, T. (2017). Intimate surveillance: Normalizing parental monitoring and mediation of infants online. Social media+ society, 3(2), 2056305117707192. |
| 88 | Leonard, N. R., Silverman, M., Sherpa, D. P., Naegle, M. A., Kim, H., Coffman, D. L., & Ferdschneider, M. (2017). Mobile health technology using a wearable sensorband for female college students with problem drinking: an acceptability and feasibility study. JMIR mHealth and uHealth, 5(7), e90. |
| 89 | Lidynia, C., Brauner, P., & Ziefle, M. (2017, July). A step in the right direction–understanding privacy concerns and perceived sensitivity of fitness trackers. In International Conference on Applied Human Factors and Ergonomics (pp. 42-53). Springer, Cham. |
| 90 | Loiselle, C. G., & Ahmed, S. (2017). Is connected health contributing to a healthier population?. Journal of medical Internet research, 19(11), e386. |
| 91 | Lupton, D. (2016). The diverse domains of quantified selves: self-tracking modes and dataveillance. Economy and Society, 45(1), 101-122. |
| 92 | Zisser, K. (2020). You want a hot body? You want a Bugatti? You better work (out): FitBit, neoliberalism, and the thin ideal. The iJournal: Graduate Student Journal of the Faculty of Information, 5(2). |
| 93 | Yang, N., van Hout, G., Feijs, L., Chen, W., & Hu, J. (2016). i-Ribbon: social expression through wearables to support weight-loss efforts. In Intelligent Environments 2016 (pp. 524-533). IOS Press. |
| 94 | Yang, H., Yu, J., Zo, H., & Choi, M. (2016). User acceptance of wearable devices: An extended perspective of perceived value. Telematics and Informatics, 33(2), 256-269. |
| 95 | Wissinger, E. (2017). Wearable tech, bodies, and gender. Sociology Compass, 11(11), e12514. |
| 96 | Wissinger, E. (2018). Blood, sweat, and tears: Navigating creepy versus cool in wearable biotech. Information, Communication & Society, 21(5), 779-785. |
| 97 | Friel, C. P., & Garber, C. E. (2020). Who uses wearable activity trackers and why? A comparison of former and current users in the united states. American Journal of Health Promotion, 34(7), 762-769. |
| 98 | Whelan, E., McDuff, D., Gleasure, R., & Vom Brocke, J. (2018). How emotion-sensing technology can reshape the workplace. MIT Sloan Management Review, 59(3), 7-10. |
| 99 | Baskaran, K., & Mathew, S. K. What Do You Fear?: A Study on User Generated Health Data and Privacy Behavior. |
| 100 | Arnold, J. F., & Sade, R. M. (2017). Wearable technologies in collegiate sports: the ethics of collecting biometric data from student-athletes. The American Journal of Bioethics, 17(1), 67-70. |
| 101 | Duru, A. (2018). Wearable cameras, in-visible breasts: intimate spatialities of feminist research with wearable camcorders in Istanbul. Gender, Place & Culture, 25(7), 939-954. |
| 102 | DiStefano, M. J. (2017). Wearable Biometric Technologies and Public Health. The American Journal of Bioethics, 17(1), 79-81. |
| 103 | Wang, C., Guo, X., Wang, Y., Chen, Y., & Liu, B. (2016, May). Friend or foe? Your wearable devices reveal your personal pin. In Proceedings of the 11th ACM on Asia Conference on Computer and Communications Security (pp. 189-200). |
| 104 | Krüger, S., & Ni Bhroin, N. (2020). Vital signs: Innovations in self-tracking health insurance and social change. Journal of Media Innovations, 6(1). |
| 105 | Ahmad, A., Rasul, T., Yousaf, A., & Zaman, U. (2020). Understanding Factors Influencing Elderly Diabetic Patients’ Continuance Intention to Use Digital Health Wearables: Extending the Technology Acceptance Model (TAM). Journal of Open Innovation: Technology, Market, and Complexity, 6(3), 81. |
| 106 | Martinez-Martin, N. (2020). Trusting the bot: Addressing the ethical challenges of consumer digital mental health therapy. In Developments in Neuroethics and Bioethics (Vol. 3, pp. 63-91). Academic Press. |
| 107 | Adamo, J. E., Bienvenu II, R. V., Dolz, F., Liebman, M., Nilsen, W., & Steele, S. J. (2020). Translation of digital health technologies to advance precision medicine: informing regulatory science. Digital biomarkers, 4(1), 1-12. |
| 108 | Torre, I., Sanchez, O. R., Koceva, F., & Adorni, G. (2018). Supporting users to take informed decisions on privacy settings of personal devices. Personal and Ubiquitous Computing, 22(2), 345-364. |
| 109 | Thornham, H. (2019). Algorithmic vulnerabilities and the datalogical: Early motherhood and tracking-as-care regimes. Convergence, 25(2), 171-185. |
| 110 | Vinnikova, A., Lu, L., Wei, J., Fang, G., & Yan, J. (2020). The use of smartphone fitness applications: The role of self-efficacy and self-regulation. International Journal of Environmental Research and Public Health, 17(20), 7639. |
| 111 | Boyd, R. L., Pasca, P., & Lanning, K. (2020). The personality panorama: Conceptualizing personality through big behavioural data. European Journal of Personality, 34(5), 599-612. |
| 112 | Seah, M. L. C., & Koh, K. T. (2021). The efficacy of using mobile applications in changing adolescent girls’ physical activity behaviour during weekends. European Physical Education Review, 27(1), 113-131. |
| 113 | Cummins, P. (2017). TBI and NFL Culture: Can Players Autonomously Refuse Biometric Monitoring?. The American Journal of Bioethics, 17(1), 75-77. |
| 114 | Asimakopoulos, S., Asimakopoulos, G., & Spillers, F. (2017, March). Motivation and user engagement in fitness tracking: Heuristics for mobile healthcare wearables. In Informatics (Vol. 4, No. 1, p. 5). Multidisciplinary Digital Publishing Institute. |
| 115 | Spil, T. A., Kijl, B., & Romijnders, V. (2019, June). The adoption and diffusion of wearables. In International Working Conference on Transfer and Diffusion of IT (pp. 31-47). Springer, Cham. |
| 116 | Twardoch-Raś, E. (2020). Somatic Narratives about Illness. Biometric Visualization of Diseased and Disabled Bodies in Art and Science Projects. Humanities, 9(1), 19. |
| 117 | Habibipour, A., Padyab, A. M., & Ståhlbröst, A. (2019). Social, ethical and ecological issues in wearable technologies. In AMCIS 2019, Twenty-fifth Americas Conference on Information Systems, Cancun, México, Augusti 15-17, 2019. Association for Information Systems. |
| 118 | Skinner, A. L., Attwood, A., Baddeley, R., Evans-Reeves, K., Bauld, L., & Munafo, M. (2017). Digital phenotyping and the development and delivery of health guidelines and behaviour change interventions. Addiction, 112(7), 1281-1285. |
| 119 | Shuren, J., Patel, B., & Gottlieb, S. (2018). FDA regulation of mobile medical apps. Jama, 320(4), 337-338. |
| 120 | Sharon, T., & Zandbergen, D. (2017). From data fetishism to quantifying selves: Self-tracking practices and the other values of data. New Media & Society, 19(11), 1695-1709. |
| 121 | Sharon, T. (2017). Self-tracking for health and the quantified self: Re-articulating autonomy, solidarity, and authenticity in an age of personalized healthcare. Philosophy & Technology, 30(1), 93-121. |
| 122 | Schüll, N. D. (2016). Data for life: Wearable technology and the design of self-care. BioSocieties, 11(3), 317-333. |
| 123 | Sanders, R. (2017). Self-tracking in the digital era: Biopower, patriarchy, and the new biometric body projects. Body & Society, 23(1), 36-63. |
| 124 | Ruckenstein, M., & Pantzar, M. (2017). Beyond the quantified self: Thematic exploration of a dataistic paradigm. New Media & Society, 19(3), 401-418. |
| 125 | Richardson, S., & Mackinnon, D. (2018). Becoming Your Own Device: Self-Tracking ChallengesIn The Workplace. Canadian Journal of Sociology/Cahiers canadiens de sociologie, 43(3), 265-289. |
| 126 | Puri, A. (2017). Acceptance and usage of smart wearable devices in Canadian older adults (Master's thesis, University of Waterloo). |
| 127 | Puri, A., Kim, B., Nguyen, O., Stolee, P., Tung, J., & Lee, J. (2017). User acceptance of wrist-worn activity trackers among community-dwelling older adults: mixed method study. JMIR mHealth and uHealth, 5(11), e8211. |
| 128 | Gooding, P., & Resnick, K. (2020). Psychiatry and law in the digital age: untangling the hype, risk and promise. |
| 129 | Preusse, K. C., Mitzner, T. L., Fausset, C. B., & Rogers, W. A. (2017). Older adults’ acceptance of activity trackers. Journal of Applied Gerontology, 36(2), 127-155. |
| 130 | Polyviou, A., Pouloudi, N., Pramatari, K., & Dhillon, G. (2021). Digital Emancipation: Are We Becoming Prisoners of Our Own Device?. In Digital Transformation and Human Behavior (pp. 165-171). Springer, Cham. |
| 131 | Pols, J., Willems, D., & Aanestad, M. (2019). Making sense with numbers. Unravelling ethico‐psychological subjects in practices of self‐quantification. Sociology of health & illness, 41, 98-115. |
| 132 | Langford, J., Poikola, A., Janssen, W., Lähteenoja, V., & Rikken, M. (2020). Understanding MyData Operators. MyData Global. https://mydata. org/wpcontent/uploads/sites/5/2020/04/Understanding-Mydata-Operators-pages. pdf. |
| 133 | Pink, S., Sumartojo, S., Lupton, D., & Heyes La Bond, C. (2017). Mundane data: The routines, contingencies and accomplishments of digital living. Big Data & Society, 4(1), 2053951717700924. |
| 134 | Perez, A. J., & Zeadally, S. (2017). Privacy issues and solutions for consumer wearables. It Professional, 20(4), 46-56. |
| 135 | Maharjan, S. M., Poudyal, A., van Heerden, A., Byanjankar, P., Thapa, A., Islam, C., ... & Hagaman, A. (2021). Passive sensing on mobile devices to improve mental health services with adolescent and young mothers in low-resource settings: the role of families in feasibility and acceptability. BMC medical informatics and decision making, 21(1), 1-19. |
| 136 | Paluch, S., & Tuzovic, S. (2019). Persuaded self-tracking with wearable technology: carrot or stick?. Journal of Services Marketing. |
| 137 | Paldan, K., Sauer, H., & Wagner, N. F. (2018). Promoting inequality? Self-monitoring applications and the problem of social justice. AI & society, 1-11. |
| 138 | Owens, J., & Cribb, A. (2019). ‘My Fitbit Thinks I Can Do Better!’do health promoting wearable technologies support personal autonomy?. Philosophy & Technology, 32(1), 23-38. |
| 139 | Emerging Issues Task Force, International Neuroethics Society. (2019). Neuroethics at 15: The current and future environment for neuroethics. AJOB neuroscience, 10(3), 104-110. |
| 140 | Will, C. M., Henwood, F., Weiner, K., & Williams, R. (2020). Negotiating the practical ethics of ‘self-tracking’in intimate relationships: Looking for care in healthy living. Social Science & Medicine, 266, 113301. |
| 141 | Nebeker, C., Lagare, T., Takemoto, M., Lewars, B., Crist, K., Bloss, C. S., & Kerr, J. (2016). Engaging research participants to inform the ethical conduct of mobile imaging, pervasive sensing, and location tracking research. Translational behavioral medicine, 6(4), 577-586. |
| 142 | Moore, P., & Robinson, A. (2016). The quantified self: What counts in the neoliberal workplace. new media & society, 18(11), 2774-2792. |
| 143 | Moore, P., & Piwek, L. (2017). Regulating wellbeing in the brave new quantified workplace. Employee Relations. |
| 144 | Mills, A. J., Watson, R. T., Pitt, L., & Kietzmann, J. (2016). Wearing safe: Physical and informational security in the age of the wearable device. Business Horizons, 59(6), 615-622. |
| 145 | Mercer, K., Giangregorio, L., Schneider, E., Chilana, P., Li, M., & Grindrod, K. (2016). Acceptance of commercially available wearable activity trackers among adults aged over 50 and with chronic illness: a mixed-methods evaluation. JMIR mHealth and uHealth, 4(1), e4225. |
| 146 | Zulueta, J., Leow, A. D., & Ajilore, O. (2020). Real-time monitoring: a key element in personalized health and precision health. Focus, 18(2), 175-180. |
| 147 | Loi, M. (2019). The digital phenotype: A philosophical and ethical exploration. Philosophy & Technology, 32(1), 155-171. |
| 148 | D’Souza, R., & De Sousa, A. (2017). Ethical Aspects of using the Digital Phenotypes of patients in psychiatric assessment. GLOBAL BIOETHICS ENQUIRY, 103. |
| 149 | Hswen, Y., Naslund, J. A., Brownstein, J. S., & Hawkins, J. B. (2018). Online communication about depression and anxiety among twitter users with schizophrenia: preliminary findings to inform a digital phenotype using social media. Psychiatric Quarterly, 89(3), 569-580. |
| 150 | Mohr, D. C., Shilton, K., & Hotopf, M. (2020). Digital phenotyping, behavioral sensing, or personal sensing: names and transparency in the digital age. NPJ digital medicine, 3(1), 1-2. |
| 151 | Loi, M., Hauser, C., & Christen, M. (2020). Highway to (digital) surveillance: when are clients coerced to share their data with insurers?. Journal of Business Ethics, 1-13. |
